# Strengthening the Cavitation Resistance of Cylinder Liners Using Surface Treatment with Electroless Ni-P (ENP) Plating and High-Temperature Heat Treatment

**DOI:** 10.3390/ma18051087

**Published:** 2025-02-28

**Authors:** Wenjuan Zhang, Hao Gao, Qianting Wang, Dong Liu, Enlai Zhang

**Affiliations:** 1 School of Mechanical and Electric Engineering, Sanming University, Sanming 365004, China; 20161134@fjsmu.edu.cn; 2 SINOMACH Intelligence Technology Co., Ltd., Guangzhou 510700, China; wqt@xmut.edu.cn; 3 ZYNP International Corporation, Industrial Cluster District, Mengzhou 454750, China; zynpliudong@163.com; 4 Henan Key Laboratory of Friction Pair Sealing Technology and Application for Cylinder Liner of Internal Combustion Engine, Mengzhou 454750, China; 5 School of Mechanical and Automotive Engineering, Xiamen University of Technology, Xiamen 361024, China; zhangel@xmut.edu.cn

**Keywords:** electroless Ni-P plating, high-temperature heat treatment, cavitation, weight loss, surface hardness

## Abstract

As internal combustion engines (ICEs) develop towards higher explosion pressures and lower weights, their structures need to be more compact; thus, the wall thickness of their cylinder liners is reducing. However, intense vibrations in the cylinder liner can lead to coolant cavitation and, in severe cases, penetration of the liner, posing a significant reliability issue for ICEs. Therefore, research on cylinder liner cavitation has attracted increasing interest. Gray cast iron is widely used in cylinder liners for its hardness and wear resistance; however, additional surface plating is necessary to improve cavitation resistance. This study developed a novel surface-modification technology using electroless Ni-P plating combined with high-temperature heat treatment to create cylinder liners with refined grains, low weight loss rate, and high hardness. The heat-treatment temperature ranged from 100 to 600 °C. An ultrasonic cavitation tester was used to simulate severe cavitation conditions, and we analyzed and compared Ni-P-plated and heat-treated Ni-P-plated surfaces. The findings showed that the combination of Ni-P plating with high-temperature heat treatment led to smoother, more refined surface grains and the formation of cellular granular structures. After heat treatment, the plating structure converted from amorphous to crystalline. From 100 to 600 °C, the weight loss of specimens was within the range of 0.162% to 0.573%, and the weight loss (80.2% lower than the plated surface) and weight loss rate at 600 °C were the smallest. Additionally, cavitation resistance improved by 80.1%. The microhardness of the heat-treated plated surface reached 895 HV at 600 °C, constituting a 306 HV (65.8%) increase compared with that of the unplated surface, and a 560 HV increase compared with that of the maximum hardness of the plated surface without heat treatment of 335 HV, with an enhancement rate of 62.6%.

## 1. Introduction

**Figure 1:**
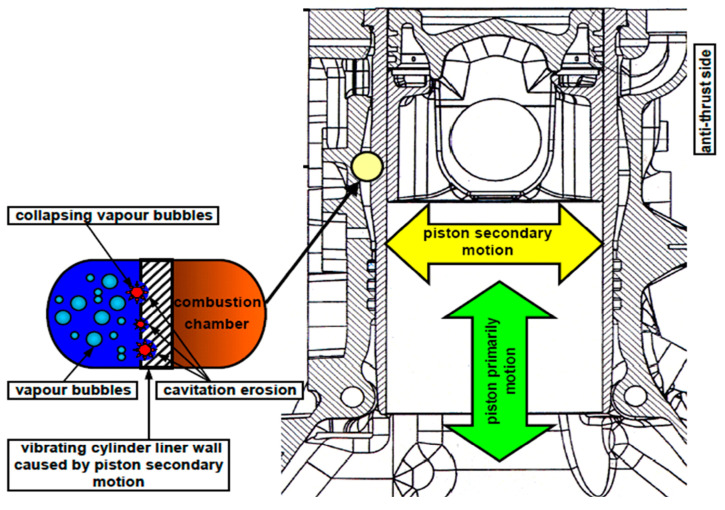
Vibration of piston cylinder liner causes coolant pressure fluctuation and bubble formation.

With the internal combustion engine (ICE) becoming lightweight and operating with high burst pressure and power, its effective pressure, speed, and specific power constantly increase, whereas the specific mass becomes increasingly small and compact. Consequently, the thickness of the ICE cylinder liner decreases. A key component of the ICE power system, the cylinder liner plays a crucial role in determining the overall quality of the engine. However, cooling water in contact with the surface of an ICE cylinder liner often causes localized aggregation of cavities, a phenomenon known as cavitation [1]. Owing to their high combustion pressures and intense piston motion, diesel engines are more susceptible to cylinder liner cavitation compared to gasoline engines, which operate at lower combustion pressures with reduced cavitation risks. Consequently, cylinder liner cavitation is one of the main reliability problems which diesel ICEs encounter [2,3,4]. Cylinder liner cavitation is typically caused by coolant cavitation induced by the vibration of the second-order motion of a piston [5,6,7], as shown in Figure 1. Due to the natural clearance between the piston and cylinder liner, variations in the lateral contact force on the piston result in side-to-side piston movement and piston rotation around the gudgeon pin, known as second-order piston motion. The mechanical impact generated by this motion is transmitted through the cylinder liner to the coolant, leading to pressure fluctuations and the formation of bubbles due to cavitation. The initial theory of bubble cavitation [8] proposed that, when pressure inside a bubble of an ideal incompressible fluid rises, it prevents the bubble walls from collapsing inward. This results in the bubble expanding, creating a pressure transient that develops into a shockwave front. The material surface is subjected to the repeated action of bubble-bursting microjets [9,10] or shockwaves, resulting in plastic deformation, fatigue and, ultimately, loss of surface quality, causing cavitation [11,12,13,14].

Severe cavitation on the surfaces of parts (e.g., cylinder liners, marine propellers, pumps, housings, and other parts that operate in a liquid environment) subjected to hydrodynamic forces can cause damage to their structure and even shorten their service life [15]. The performance of a surface plays a crucial role in determining the cavitation resistance of components. To improve the cavitation resistance of metals or alloys and extend their lifespan, researchers have explored various methods for surface modification of engineering alloys, such as laser surface modification technology [2,16], laser impact strengthening [17,18,19,20], and laser surface cladding [21,22,23]. Among these methods, laser surface strengthening is expensive, complicated, and costly, and microstructural changes can easily occur, owing to the high temperatures generated by laser ablation of the substrate surface [24,25,26]. Other surface technologies, such as anodic oxidation process [27,28], nano-ceramic coated surfaces [29,30,31], electro-discharge machining [32,33], double cathode discharge [34,35], plasma nitriding [36,37,38,39], gas nitriding [40,41,42], ion implantation [43], and surface chromatization [44], have been investigated for coatings, stainless steel, aluminum alloys, and other alloys and substrate materials to increase cavitation resistance. However, anodizing is performed in a specific electrolytic solution, which involves the risk of environmental pollution. Surface treatments, like nitriding and chroming, often involve extended processing times, which can result in the formation of coarse grains and cause undesirable deformation of the components. Therefore, improving the mechanical properties of surfaces without polluting the environment, while ensuring economic viability, is an innovative way to avoid these defects. In parallel, sacrificial protection strategies based on metal salt additives (e.g., nitrites, molybdates) in engine coolants have been widely adopted in industrial practice. These additives chemically adsorb onto liner surfaces, forming transient protective films that mitigate cavitation damage by absorbing impact energy through sacrificial degradation [15]. While such methods are cost-effective and easy to implement, their efficacy is constrained by strict coolant conditions (e.g., pH, temperature) and necessitates continuous replenishment [45].

Compared with the above-mentioned surface-enhancement techniques, electroless Ni-P (ENP) plating is an autocatalytic reaction that is widely used as a simple, economical, environmentally friendly, and effective surface-treatment technique, because it does not require an applied current or anode; the plating is uniformly organized, friction- and corrosion-resistant, and does not use hazardous substances, such as cyanide [46,47]. Boakye et al. [48] developed an ENP biphasic coating containing polytetrafluoroethylene. Three types of polytetrafluoroethylene Ni-P biphasic coatings with low, medium, and high contents were deposited on the surface of mild steel to study its mechanical and friction properties. The results revealed that the biphasic coating provided the highest wear protection for the substrate under the lowest load. However, as the load and number of sliding cycles increased, leading to a higher wear rate of the coating, the lubrication performance of the medium-content biphasic coating improved by 79%. The wear resistance of the high-content Ni-P coating showed a notable improvement, reaching 8.3 × 10^4^ m/mm^3^, compared to the low and medium-content coatings. Park et al. [49] utilized an electroless nickel plating method to apply a coating to the surface of gray cast iron. They examined how different pH values (4.0, 4.8, 5.6, and 6.4) of the plating solution influenced the corrosion resistance of Ni coatings and their behavior under cavitation damage in seawater. They observed that the P content in Ni coatings decreased with increasing pH, the coatings transformed from amorphous to crystalline structures, and the coating rate and matrix organization strongly influenced the corrosion resistance of the coatings. In addition, the authors investigated the corrosion resistance of ENP- and Ni-plated surfaces in seawater [50,51]. Beyond process parameters, the intrinsic composition of the coating (specifically the Ni-P content) has also been identified as a critical determinant of performance. Studies indicate that high-phosphorus (10–14 wt%) coatings tend to form amorphous structures, exhibiting superior corrosion resistance and uniformity but higher brittleness. Medium-phosphorus (6–9 wt%) coatings achieve corrosion resistance and abrasion performance suitable for most industrial applications, whereas low-phosphorus (3–5 wt%) coatings predominantly crystallize, offering higher hardness and wear resistance at the expense of reduced corrosion resistance [52,53,54]. Arumugam et al. [55] added an ionic surfactant and nano-Al_2_O_3_ to a Ni-P plating bath to coat a base aluminum alloy. The Ni-P coating of the aluminum alloy was investigated using 10- and 20-N loads, applied using a disc wear test. The results showed that an Ni-P coating with the addition of an ionic surfactant activator significantly improved the wear resistance of the aluminum alloy specimens. Similarly, Vetrivezhan et al. [56] employed an ENP coating with the addition of SiC for the surface treatment of an aluminum alloy. The microstructure and microhardness of the coatings were analyzed by selecting three pH ranges (4–5, 6–7, 8–9) and temperatures of 70, 85, and 90 °C, which improved the microhardness in order to strengthen surface wear resistance. The test results indicated that the highest microhardness of the coating (650 HV) occurred at a pH range of 6–7 and a temperature of 85 °C. Reis et al. [57] coated the surface of copper–beryllium alloys using ENP plating and heated the coated specimens to 200 °C for 24 h. The cross-sectional microhardness distributions of the heat-treated sections were measured to evaluate the hardness of the coatings and substrates. They found that the combination of Ni-P coating and heat treatment significantly increased the surface hardness of uncoated copper–beryllium alloys, raising it from 340 to 997 HV. Additionally, the wear coefficient decreased from 3.03 × 10^−6^ to 2.04 × 10^−6^ mm^3^/N·m. In the aforementioned studies, ENP plating surface technology is mostly combined with low-carbon steel, aluminum alloy, copper–beryllium alloy, and other materials, focusing on the performance of the coating in terms of corrosion resistance and wear resistance. ENP plating has been applied to gray cast iron, but research in the seawater environment primarily explores corrosion resistance and cavitation resistance, i.e., the use of ENP plating combined with high-temperature heat treatment surface technology applied to cast iron, focusing on the study of cavitation resistance, is rarely reported. Moreover, owing to its combined advantages of high strength and wear resistance, gray cast iron is commonly utilized in internal combustion engine cylinder liners [58,59]. The surface of gray cast iron cylinder liners are prone to cavitation, which affects their durability in ICEs. Therefore, given that ENP plating is one of the major surface coating technologies being developed in today’s industry [51], it is essential to enhance the cavitation resistance of cast iron alloy cylinder liners through the application of ENP plating in combination with high-temperature heat treatment as a surface modification technique.

In this study, an innovative combination of ENP plating and high-temperature treatment was used to study the cavitation performance of cylinder-liner gray cast iron. The differences in the cavitation performance of uncoated, Ni-P-coated, and Ni-P-coated specimens after high-temperature treatment were analyzed. Key indexes, such as cavitation morphology, weight loss, and surface microhardness of Ni-P-coated specimens after high-temperature treatment, were further analyzed to study the control mechanism of cavitation damage in depth and provide a theoretical basis and practical guidance for research and application in related fields.

## 2. Material and Methods

### 2.1. Material

**Figure 2:**
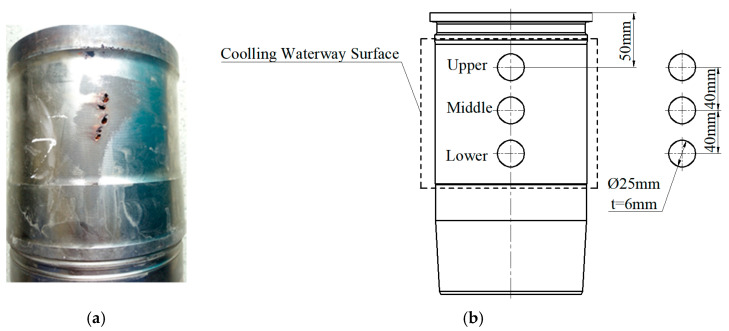
Unplated cylinder liner cavitation location and plated cylinder liner specimen location dimensions: (**a**) unplated cavitation, (**b**) ENP plated specimen location and geometry.

Cylinder-liner gray cast iron was chosen as the test material, with its chemical composition provided in Table 1. The vibration impact force revealed that cavitation in the cylinder liner initiated from the waterway and upper annular band regions. During engine operation, the piston exerts the greatest force and experiences the most unstable movement at the TDC and BDC. This leads to vibration in the cylinder liner, making cavitation more likely to occur in areas near the thrust force, as illustrated in Figure 2a [60].

According to the location of the cylinder liner prone to cavitation, the outer surface area of the cooling water channels of the cylinder liner was taken as the experimental object, and the specimen geometry was a disk of Φ25 mm and 6 mm thickness, as shown in Figure 2b. Thirty-six cylinder liner specimens without plating and with Ni-P plating were obtained from the upper, middle, and lower positions for the ultrasonic cavitation test.

### 2.2. ENP Plating Surface Treatment and Ultrasonic Cavitation Experiment

The specimens were polished, descaled, degreased, activated, and then immersed in a plating bath. In this experiment, ENP plating was applied to treat the outer surfaces of the specimens under the following composition and process conditions: sodium hypophosphite (25 g/L), glycine (30 g/L), sodium acetate (20 g/L), phosphorus content (45 g/L), and lactic acid (30 mL/L). The plating solution had a temperature of 80 °C and a nickel sulfate NiSO_4_ as the nickel source concentration of 5 g/L, chosen for its ability to ensure homogeneous phosphorus distribution and stable deposition rates in acidic conditions (pH = 5) [61,62]. The plating time was set to 60 min, resulting in a plating layer thickness of approximately 20 μm. After electroplating, the Ni-P cylinder liner specimens were placed in a box-type resistance furnace (RJX-2.5-10 Henan Aufeda Instrument, Zhengzhou, China) and heat-treated at temperatures of 100, 200, 300, 400, 500, and 600 °C, with a 2-h holding time. Post-deposition heat treatment further modulated the crystallinity and phase distribution (e.g., Ni_3_P precipitation) [52]. Afterward, the specimens were air-cooled to room temperature.

**Figure 3:**
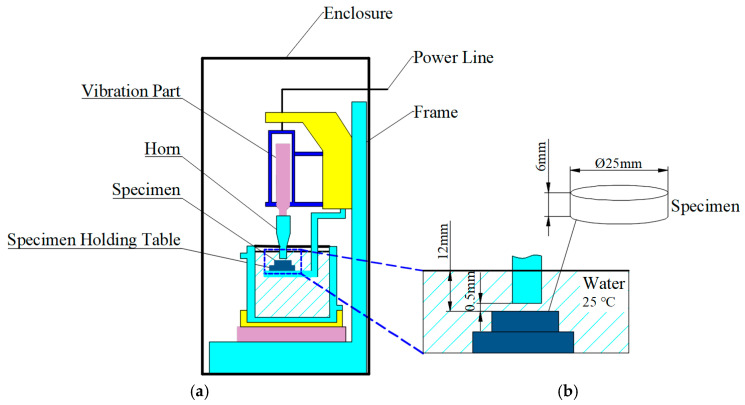
Ultrasonic cavitation test device: (**a**) Schematic diagram of the test device; (**b**) Partial enlargement.

An ultrasonic cavitation tester (YP5020-6D, Hangzhou Successful Ultrasonic Equipment Co., Hangzhou, China) was used to accelerate the simulation of severe cavitation conditions of the unplated, Ni-P-plated, and heat-treated specimens. The power of the cavitation testing machine was 1 KW, with a vibration frequency of 20 kHz and an amplitude of 50 μm. The test temperature was set to 25 °C, and the test solution medium was pure water with a liquid depth of 100 mm. The clearance between the test specimen and the tip of the vibration head was 0.5 mm, with the upper surface of the specimen situated at a depth of 12 mm from the liquid surface. The test time was set to 15 h. Based on the requirements of ASTM G32-10 [63], a static sample-testing method was used: the specimen was fixed on a table in the container liquid, and the top of the vibration component-mounted amplitude rod created cavitation bubbles that impacted the upper surface of the specimen to simulate the effect of cylinder liner cavitation, as shown in Figure 3. When the test was completed, the specimens were removed for cleaning and drying, and the cumulative weight loss was determined using an electronic balance. The test was conducted three times, and the average values were calculated to ensure reliability of the results.

### 2.3. Characterization Equipment

An EDS (X-MAX Oxford Instruments, Oxford, UK) was used to analyze the elements and contents of the cavitation micro-regions of the unplated, Ni-P-plated, and heat-treated plated specimens. A Tungsten filament SEM (EVO18, Zeiss, Jena, Germany) was employed to observe the cavitation surface morphology of the unplated, Ni-P-plated, and heat-treated plated specimens. A metallographic microscope (DM2000, Leica, Wetzlar, Germany) was used to observe the surface cavitation microstructure of the Ni-P plating. The cavitation surface morphology of the specimens and microstructure of the surface cavitation of the Ni-P plating were observed using the Leica metallographic microscope. The specimens were weighed before and after the cavitation test using an electronic balance model MS105DU (Mettler Toledo MTCN Limited Company, Hong Kong, China). The rate of weight loss was calculated as follows:

loss rate = (W_0_ − W_1_)/W_0_ (1)

where W_0_ and W_1_ are the weights before and after the test, respectively. A microhardness tester (TH765A, Beijing Time High Technology, Beijing, China) was used to determine the microhardness of the unplated and heat-treated Ni-P plating with a set load of 0.1 kg. Five points on the specimen were randomly selected for measurements, and the average values were labeled as HV hardness.

## 3. Results and Analyses

### 3.1. Surface Morphology and Energy Spectrum Analysis

#### 3.1.1. Unplated Surface Morphology and Energy Spectral Analysis

**Figure 4:**
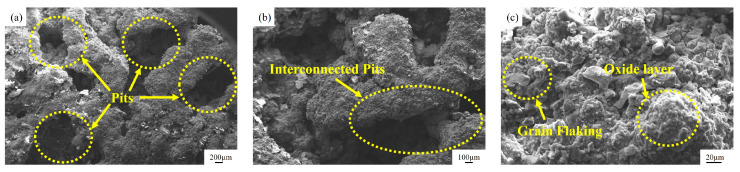
The surface topography of the unplated specimens after cavitation testing: (**a**) 50×, (**b**) 100×, (**c**) 500×.

**Figure 5:**
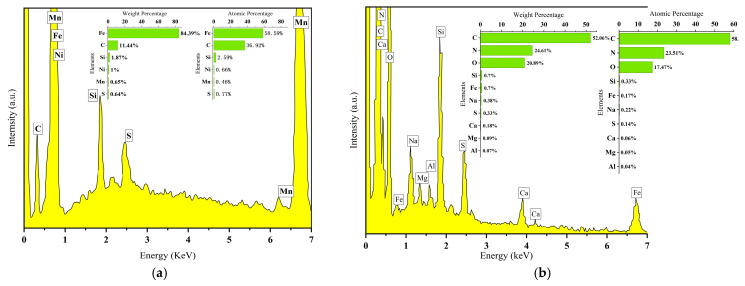
The energy spectra of the uncavitated and cavitated regions of the unplated specimens: (**a**) region without cavitation, (**b**) cavitation region.

Figure 4 shows the surface topography of the unplated specimens after cavitation testing. Magnifications of 50× and 100× showed the presence of deeper, interconnected pits on the surfaces of the specimens, as shown in Figure 4a,b. Grain exfoliation on the surface of the specimen is observed in Figure 4c, where the surface of the cavitation-pitted grains is covered with a layer of oxide film and oxides with non-uniform grain sizes and inconsistent pit depths. Figure 5 compares the energy spectra of the uncavitated and cavitated regions of the unplated specimens. Figure 5a indicates the region where no cavitation occurred in the unplated specimen. This region consists of 36.92% C and 58.59% Fe atoms, respectively. Therefore, this composition is more favorable. Si, S, Mn, and Ni element atoms accounted for 2.59%, 0.77%, 0.46%, and 0.66%, which are normal compositional contents of the cylindrical liner specimen. The EDS of the cavitation region showed that elements such as N, O, Na, Mg, Al, Si, S, and Ca were detected in the cavitation region of the specimen with atomic percentages of 23.51%, 17.47%, 0.22%, 0.05%, 0.04%, 0.33%, 0.14%, and 0.06%, respectively, the atomic percentage of elemental Fe being 0.17%, which was 58.42% less than the percentage of elemental atoms in the area without cavitation (Figure 5b). The cavitation region of Na, Mg, Al, Si, S, and Ca elemental oxides produced more impurities than those in the area without cavitation, and the surface of cast iron inside the cavitation pits analyzed using the energy spectrum was covered by a large number of oxidized layers and compounds, such as MgO, Al_2_O_3_, SiO_2_, and FeS_2_. This occurs because the burst of the cavitation bubbles at an early stage impacts the outer surface of the cylinder liner, leading to the formation of pits. Cavitation pits gradually expand on the outer surface of the cylinder liner to form larger pits, which easily form dead water or vortices, thereby forming an impurity with the substrate material oxide film and oxide layer, intensifying the depth of cavitation. When the outer surface of the cylinder liner is not plated, cavitation area impurities, and Fe in the matrix is more likely than other elements in the coolant to form a primary cell; electrochemical corrosion occurs, accelerating the cavitation electrochemical corrosion rate. The surface chemical reaction formula is

O_2_ + 2H_2_O + 4^e−^→4OH^−^ (2)

Fe is oxidized and loses electrons to form Fe^2+^ in the reaction equation:

Fe + 2^e−^→Fe^2+^ (3)

Fe^2+^ reacts with OH- to form Fe(OH)_2_, which changes to Fe(OH)_3_ and Fe_2_O_3_ according to the following reaction:

4Fe(OH)_2_ + O_2_ + 2H_2_O→4Fe(OH)_3_ (4)

2Fe(OH)_3_→Fe_2_O_3_ + 3H_2_O (5)

#### 3.1.2. Surface Morphology and Energy Spectrum Analysis of Ni-P Plated Layer

**Figure 6:**
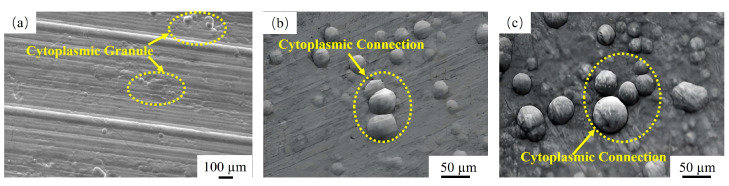
SEM of Ni-P plating in plated state and after heat treatment, (**a**) plated state, (**b**) 200 °C, (**c**) 300 °C.

**Figure 7:**
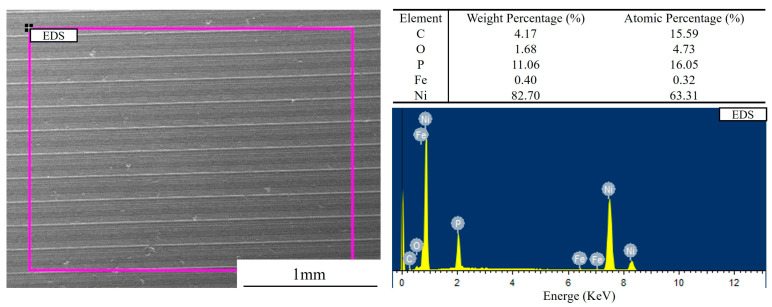
EDS of Ni-P plating in plated state.

Figure 6 presents the SEM of the Ni-P-plated specimens before the cavitation test was performed. It is observed that the surface of the specimen in the plating state at this time was composed of cytoplasmic micro-convex granules, and the plating surface tended to be flat, without the appearance of defects, such as holes, voids, and cracks. Compared with the plated specimens, Figure 6b,c present the surface morphology of the specimens after heat treatment at 200 and 300 °C for 2 h. The surface morphologies of the plated specimens after heat treatment at 200 and 300 °C are illustrated in Figure 6a,b. The crystalline materials on the surface of the plated layer after heat treatment were gradually connected, and the increase in temperature caused the growth of crystalline micro-convex particles to inhibit each other, such that the grains are arranged more closely with each other, the crystallization was more detailed and uniform, and the densification was higher. As evidenced in Figure 7, the plated example exhibits a P content of 11.06 wt%, corresponding to the high-phosphorus (HP) classification. The elevated phosphorus concentration in the HP composition induces significant lattice distortion within the nickel matrix, promoting the formation of an amorphous microstructure. Critically, this amorphous phase lacks crystalline defects, such as grain boundaries and dislocations [53].

**Figure 8:**
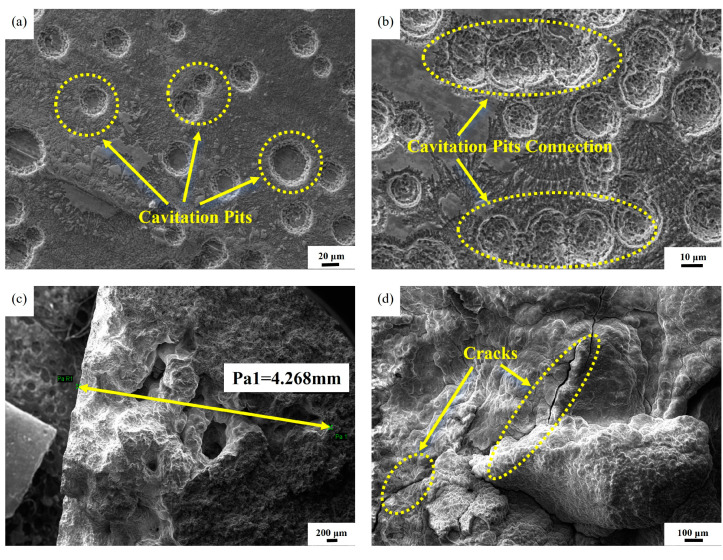
SEM of cavitation of Ni-P plated specimens without heat-treatment; (**a**) initial cavitation pit; (**b**) cavitation pits connection; (**c**) cavitation pit size; (**d**) cavitation cracks.

Ultrasonic cavitation tests were performed on Ni-P plated cylinder liner specimens. Figure 8 depicts the surface topography of the Ni-P plated cavitation without heat treatment. The cavitation increased the lateral size of the pits from the initial to final stages and progressed to the depth. Initial cavitation had a circular pit shape, spreading from the center of the circle in all directions, as shown in Figure 8a. With increasing time of the cavitation test, the cavitation area and depth gradually increased, making individual cavitation pits indistinguishable and interconnected, exhibiting one or several connected large pits with uneven heights, as presented in Figure 8b. Figure 8c indicates that the depth of the cavitation pit deepened, and more black holes appeared inside, with the transverse size of black holes reaching 4.268 mm. Black holes were formed by graphite detachment, indicating that the plating layer had already been penetrated by cavitation to reach the body of the cylinder liner specimen at this time. Figure 8d shows that, for the plated specimen subjected to cavitation test, cracks appeared on the surface of gray cast iron affected by cavitation. The Ni-P plating cavitation test simulated cylinder-liner vibration intensity, cylinder-liner outer surface coolant (cooling water) to form bubbles, bubble rupturing after shocking the cylinder-liner external surface, and cavitation formation. Therefore, the initial surface of the cavitation was mostly a regular round pit. However, with time, the size of the cavitation pits gradually increased, and the depth increased, forming a continuous section of cavitation pits of varying depths and cracks. If graphite was exposed in the plating, the bubbles would erode into the matrix material.

**Figure 9:**
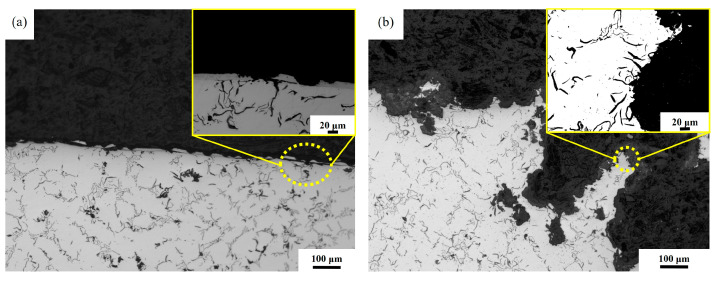
Metallographic microstructure of the Ni-P plated section without heat treatment: (**a**) cavitation in the plated section, (**b**) cavitation penetrating the plating.

Figure 9a,b depicts the metallographic microstructure of Ni-P plated specimens after cavitation. Only a small portion of plating was cavitated to expose the substrate of the specimen. Nevertheless, the cavitation did not completely penetrate the specimen substrate. As shown in Figure 9a, the cavitation started from the surface plating part, and when the surface plating was cavitated off, the cylinder liner body was exposed. If bare graphite in the cylinder liner body, such as crack defects, existed, the cavitation started from the bare graphite part. It gradually expanded to the inside of the plating to form cavitation pits. If no graphite was exposed on the outer surface of the liner, cavitation followed the surface of the liner until surface defects, such as exposed graphite or slag pores, were observed. Figure 9b presents the internal organization of the cavitation pits. Under the impact of constant coolant water flow and bubble burst, cavitation started from the exposed graphite and spread to form deeper and wider cavitation pits. In severe cases, even the entire cylinder liner wall could be penetrated by cavitation to constitute a perforation.

**Figure 10:**
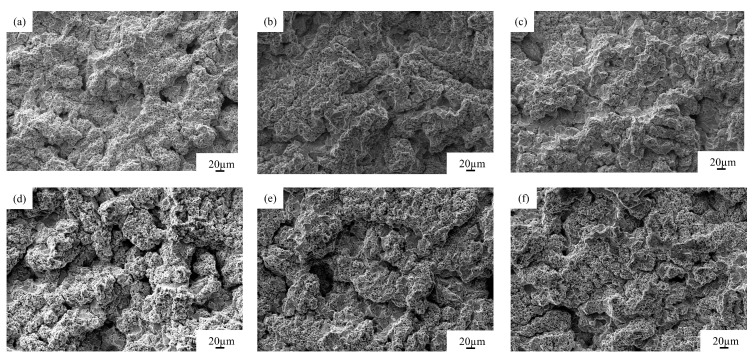
SEM of cavitation of Ni-P-plated after different heat treatments: (**a**) 100, (**b**) 200, (**c**) 300, (**d**) 400, (**e**) 500, and (**f**) 600 °C.

Figure 10 shows the SEM images of Ni-P coated specimens subjected to cavitation after heat treatment at temperatures of 100, 200, 300, 400, 500, and 600 °C. As the heat-treatment temperature rose, the surface morphology of the plated layer altered, showing signs of grain spalling. The surface of specimens exhibited microporosity and slight grain exfoliation at 100 °C, grain exfoliation was more obvious at 200 °C, significant grain exfoliation and microcracks appeared on the surface at 300 and 400 °C, and fewer plated particles were exfoliated on the surface at 500 and 600 °C compared with 400 °C after heat treatment. The weakest surface of the plated layer was repeatedly plastically deformed until work-hardening under the effect of alternating impact stress of the irregular rupture of test bubbles. Compared with the unplated specimen (Figure 4), the plating microstructure surface grains were fine and dense after heat treatment. The inter-grain bonding surface had micro-fine grain boundaries, which improved the toughness and plasticity of the plating, buffering the bubble impact stress, which caused the cavitation fatigue cracks to expand at a reduced rate.

**Figure 11:**
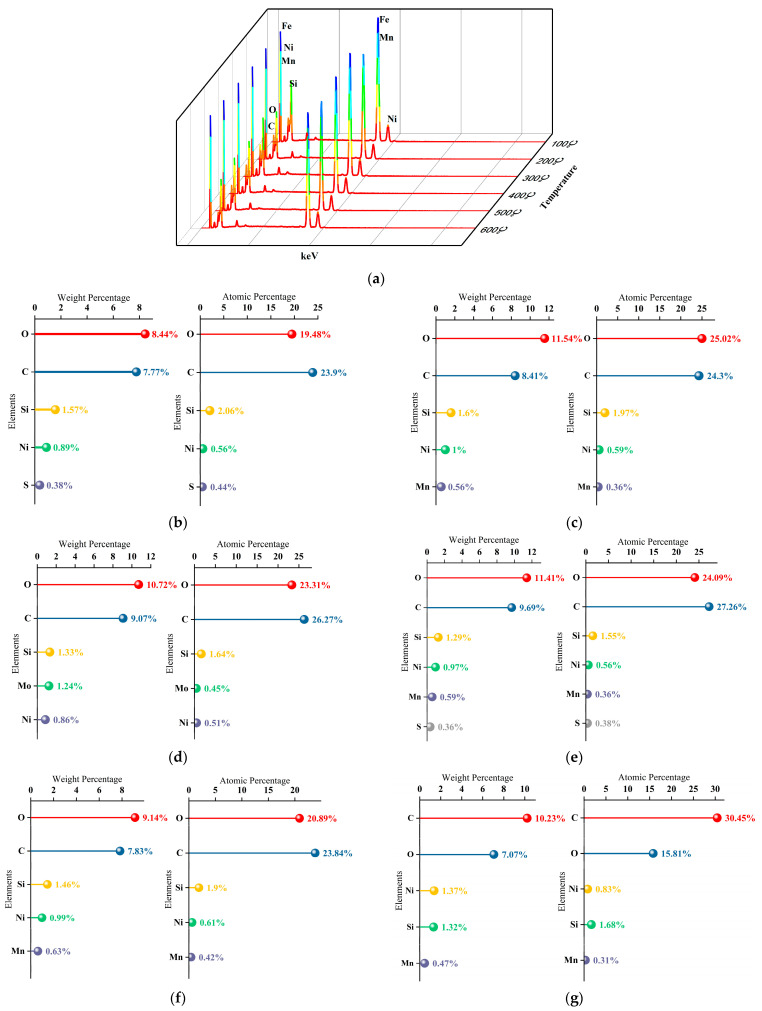
EDS and percentages of elemental compositions of plated layers at different temperatures, (**a**) EDS, (**b**) 100 °C, (**c**) 200 °C, (**d**) 300 °C, (**e**) 400 °C, (**f**) 500 °C, (**g**) 600 °C.

Figure 11 shows the EDS results for the plated surfaces after various treatments. From 100 to 600 °C, the primary elements in the plated specimens were O, C, Si, Ni, S, Mn, and Mo, except Fe. The average weight of Ni was 1.01%, with an atomic percentage of 0.61%. The percentage of elemental content in Figure 11 was similar to the composition of the area in Figure 5a, which had no cavitation. The plated surfaces of the specimens after heat treatment exhibited finer grains than the surfaces of the unplated specimens, and their elemental compositions were similar. The findings indicated that heat treatment of the Ni-P plated specimens was conducive to eliminating residual H atoms in the base alloy, relaxing internal stresses, and improving the bond between the plating and the matrix. In cavitation tests, corrosion in the later stages generally occurs at the free surface or with plating defects. When the plating completely covered the surface of the metal substrate, the corrosive medium penetrated the plating along the pits or cracks occurring on the surface of the plating, in order to electrochemically interact with the surface of the metal substrate.

### 3.2. Weight Loss Analysis

**Figure 12:**
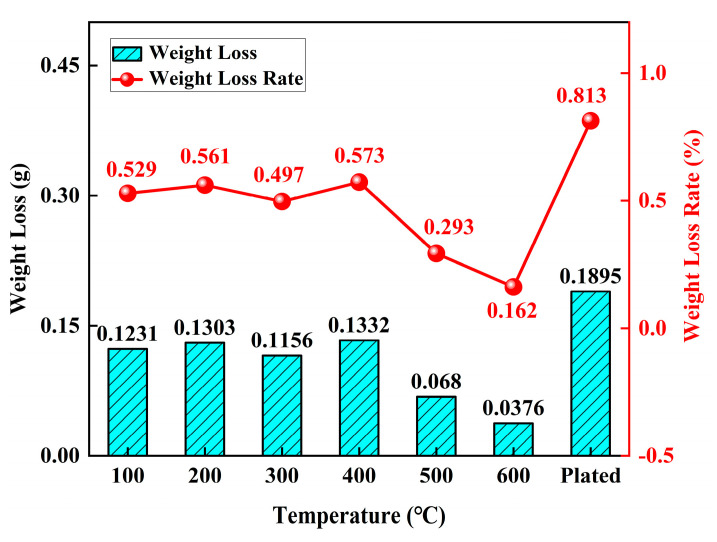
Variation in the weight loss and weight loss rate of plated cylinder liner with different temperature heat treatments.

Figure 12 illustrates the variation in and rate of weight loss for heat-treated and untreated Ni-P plating. From 100 to 400 °C, the weight loss of the plated specimens was 0.1231, 0.1303, 0.1156, and 0.1332 g, and the weight loss rates were 0.529%, 0.561%, 0.497%, and 0.573%, respectively. It was observed that the variation and rate of weight loss in the specimens were similar within the temperature range of 100 to 400 °C. The weight loss and weight loss rate of the specimens reached the highest values at heat treatment of 400 °C, at 0.1332 g and 0.573%, respectively. The weight loss and weight loss rate gradually decreased from 400 to 600 °C and reached the minimum values at 600 °C, at 0.0376 g and 0.162%, respectively. The weight loss and loss of Ni-P plating without heat treatment reached maximum values of 0.1895 g and 0.813%, respectively. The weight loss at 600 °C was 80.2% lower than that in the plated state, and the cavitation resistance improved by 80.1%, which is 59.6% lower compared to the 3 h weight loss of 0.060 g [49] for electroless nickel plating cavitation.

The data presented in Table 2 indicate that the weight loss of specimens in both the plating state and after Ni-P heat treatment was comparable, ranging from 0.0376 to 0.1895 g, with weight loss rates between 0.162% and 0.813%. The specimens in the plating state exhibited the highest weight loss and weight loss rate, while the smallest values were observed at 600 °C. According to the cavitation test, the deepest cavitation depths of the heat-treated Ni-P plating at 400 and 600 °C were 25.956 and 7.345 μm, respectively, and that of the plated state was 36.831 μm. The time taken for cavitation to reach a depth of 100 μm (t100) in the heat-treated plating (12,240 min) was significantly longer than that in the plated state (2441 min). Thus, the heat-treated Ni-P plating effectively delayed the appearance of cavitation. Figure 12 and Table 2 show that, under the same cavitation conditions and at the same time, heat-treated substrate and plating material tissue exhibit grain refinement, and Ni-P plating cavitation resistance is highly correlated to the crystallization degree during the plating heat treatment [59]. The dominant failure mechanisms of the ENP plating under cavitation conditions are fatigue and plastic deformation, with corrosion playing a secondary role [11]. Moreover, Ni-P plating exhibits an amorphous structure in the plating state, and the internal arrangement of atoms is disordered. No grain boundaries, dislocations, or other defects occur, and the material is completely isotropic and homogeneous [53]. Heat treatment transforms the coating’s microstructure from amorphous to crystalline, enhancing fatigue resistance, reducing plastic deformation, and mitigating corrosion effects. As the heat treatment temperature increases, the Ni and Ni_3_P phases precipitated in the plating layer play a diffuse strengthening role [54]. Hence, the heat-treated Ni-P plating cavitation pits are shallower, and the initial cavitation point lags behind the cavitation of the plated specimen, which effectively improves the cavitation resistance of the surface of the plated specimen.

### 3.3. Surface Microhardness

**Figure 13:**
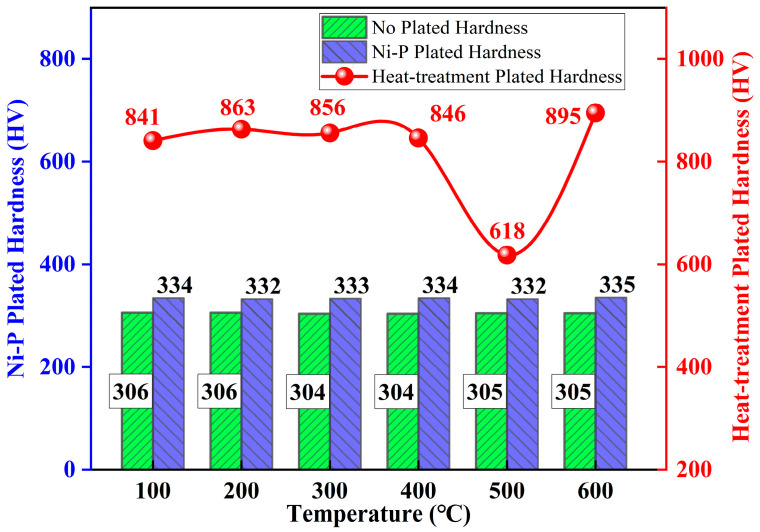
Comparison of hardness of heat-treated Ni-P plated and unplated specimens.

**Figure 14:**
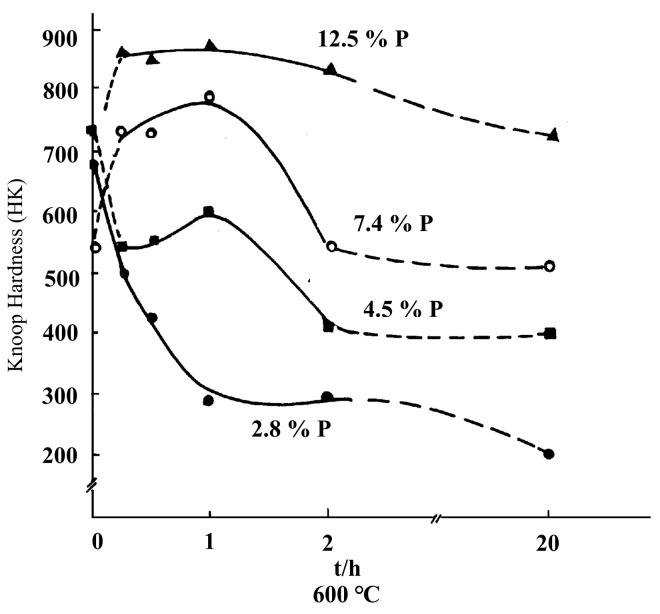
Effect of phosphorus content heat treatment time on the hardness of plated layer [65].

Figure 13 shows the microhardness values of the Ni-P plating, heat-treated Ni-P plating, and unplated samples. Each specimen was measured at five different points, and the average value was taken as the surface microhardness (HV). In both the non-Ni-P-plated and Ni-P-plated states, the microhardness of the outer surfaces did not change significantly with temperature, with average hardness values of 305 ± 1 HV for the unplated state and 333 ± 2 HV for the Ni-P-plated state. The microhardness of the Ni-P plating after heat treatment was significantly higher than that of the unplated and non-heat-treated plating, whereas hardness was also influenced by temperature, and the microhardness of the specimens at 100–600 °C was 841 ± 7, 863 ± 8, 856 ± 12, 846 ± 5, 618 ± 7 and 895 ± 9 HV, respectively. Between 100 and 400 °C, hardness fluctuated with increasing temperature, and the change in hardness value was not apparent, within the range 841–863 HV. At the beginning of the heat treatment at 100 °C, a high internal stress was observed in the Ni-P coating, and the hardness value was 841 HV. As the temperature increased to 200 °C, the internal stress in the plating gradually disappeared, and the hardness increased to 863 HV. At 300 and 400 °C, the hardness decreased slightly to 846 HV owing to the weakening effect of the release of internal stress, and the precipitation of the Ni_3_P phase began to produce a diffuse strengthening effect on the plating. The slight decrease in hardness to 846 HV at 300 and 400 °C was due to the weakening of the internal stress relief effect and the precipitation of the Ni_3_P phase, which began to have a diffuse strengthening effect on the plating. Still, this strengthening was insufficient to counteract the decrease in hardness caused by the internal stress relief. At 500 °C, with the plating from the amorphous state or nanocrystalline gradually transitioning to the microcrystalline state of and with gradually increasing grain size, the particle dispersion strengthening effect gradually weakened, and the hardness decreased. At 600 °C, owing to the phosphorus-containing supersaturated Ni-P solid solution being in a thermodynamically unstable state [64], the continuous diffusion and aggregation of P atoms occurred as the temperature increased. This increased the degree of material lattice distortion. When the diffusion of P atoms on the crystal surface of the nickel crystal reached the number of P atoms that could decompose the supersaturated solid solution and precipitate a certain amount of the second phase of Ni_3_P, a significant precipitation hardening effect occurred, and the hardness of the plating reached its maximum value (895 HV). The microhardness of the heat-treated Ni-P plating was always much higher than the surface hardness of the unplated and unheated-treated plating, for which the maximum hardness of the heat-treated plating (895 HV) was 588 HV higher than that of the unplated plating (306 HV), and the maximum hardness increase rate was 65.8%. Simultaneously, the maximum hardness of the plated surfaces without heat treatment increased to 560 HV, compared with 335 HV, constituting an increase of 62.6%. The surface microhardness of electroless nickel plated specimens at five stabilizer concentrations was measured at 767, 771, 849, 895 and 604 HV [49]. Additionally at temperatures ranging from 70 to 90 °C, the surface microhardness of the specimens was found to be 550, 580, 620, 650, and 655 HV, respectively [50]. The enhancement of hardness in ENP through high-temperature treatment is synergistically influenced by two mechanisms: precipitation hardening driven by phosphorus and modulation of the stress state through heat treatment. As illustrated in Figure 14 [65], high-phosphorus (HP) coatings exhibit superior hardness compared to their low-phosphorus (LP) counterparts. The analysis indicates that, at 600 °C, HP coatings develop stable Ni-Ni_3_P composite structures, where the dispersion of Ni_3_P phases contributes significantly to hardening. In contrast, LP coatings, which are primarily composed of the Ni phase, experience excessive grain coarsening and a consequent degradation in hardness. Importantly, this microstructural evolution (from amorphous to crystalline) not only enhances the coating’s hardness but also fundamentally alters its internal stress characteristics.

Internal stresses are inherently present in coatings and have a critical impact on interfacial stability. Tensile stress can lead to coating delamination, cracking, or blistering, while compressive stress significantly improves the adhesion between the coating and substrate [66]. Research indicates that the characteristics of coating stress (type: compressive or tensile) are influenced by multiple factors, including phosphorus content, substrate material, heat treatment processes, and plating bath composition [66].

The total coating stress (σ_T_) is comprised of intrinsic stress (σ_i_) and thermal stress (σ_H_), expressed as σ_T_ = σ_i_ + σ_H_. The intrinsic stress (σ_i_), which is typically tensile, arises from lattice mismatch during the deposition of amorphous Ni-P coatings [46,53]. Thermal stress (σ_H_) results from the difference in the coefficient of thermal expansion (CTE) between the coating and substrate during thermal cycling [67]. The CTE of gray cast iron, used for cylinder liners, increases significantly with temperature, ranging from 9.6 × 10^−6^/K at room temperature to 13.5 × 10^−6^/K at elevated temperatures. The CTE of ENP coatings decreases with increasing phosphorus content: LP (~12 × 10^−6^/K) > MP (11~12 × 10^−6^/K) > HP (~10 × 10^−6^/K). Heat treatment transforms amorphous Ni-P coatings into crystalline structures with Ni_3_P precipitates, resulting in a volumetric shrinkage of 4–6% [52,54]. This phase transition generates localized compressive stress, effectively counteracting the initial tensile stress. Due to the greater CTE mismatch (ΔCTE = substrate CTE—coating CTE) in HP coatings, compressive stress predominates in their residual stress state following high-temperature heat treatment.

Heat treatment systematically reduces residual stress in ENP coatings by facilitating the transition from an amorphous to a crystalline structure [46,47]. This structural reorganization not only enhances coating hardness and fatigue resistance [37,53] but also promotes the formation of tougher crystalline phases at elevated temperatures (e.g., 600 °C), thereby suppressing plastic deformation and the initiation of fatigue cracks [13,14]. These findings demonstrate that the ENP heat treatment strategy synergistically optimizes both the stress state (compressive-dominated) and the microstructure (dispersion of Ni_3_P precipitates), effectively addressing failure related to residual stress and mechanical deficiencies and significantly improving the durability of cylinder liners.

### 3.4. Comparison of ENP + Heat Treatment with Other Surface Modification Technologies

The ENP + heat treatment method exhibits distinct advantages and limitations when compared to other surface modification technologies, such as laser alloying and arc spraying. The anti-cavitation performance of ENP + heat treatment plating is influenced by the synergistic mechanisms of amorphous to crystalline transformation, Ni_3_P precipitation hardening, and compressive stress [52,54,65]. In contrast, laser alloying utilizes a high-energy laser beam to melt the substrate surface, along with alloying elements (such as ceramics or metals), achieving excellent surface hardness and wear resistance due to rapid solidification and fine microstructure [16,17,18]. For instance, laser surface cladding on stainless steel or tungsten carbide coatings can achieve a microhardness exceeding 1200 HV [21,22,23], which surpasses the 895 HV obtained in this study with ENP + 600 °C heat treatment. However, laser-based methods require expensive equipment and precise parameter control, and the substrate is at risk of deformation or cracking due to localized high heat input [24,25,26]. In contrast, ENP plating is a low-temperature, non-line-of-sight process that can uniformly coat complex geometries without thermal deformation, making it more economical and suitable for mass production. Additionally, the amorphous structure of ENP and the subsequent thermally induced crystallization (dispersion strengthening due to Ni_3_P phase precipitation) provide a unique combination of hardness and corrosion resistance, whereas laser alloyed coatings often exhibit residual tensile stress, which negatively impacts fatigue resistance [16,24].

Arc-sprayed coatings, such as those deposited via thermal spraying, exhibit a high deposition rate and are compatible with various materials, including ceramics and metals [33]. These coatings are suitable for large-area applications; however, they rely on the mechanical bonding of molten particles, which can lead to issues such as porosity (5–15%) and weak interfacial bonding. This necessitates post-treatment methods, such as remelting, to enhance density [33]. In contrast, ENP combined with heat treatment can produce metallurgically bonded coatings that are free of porosity. The heat treatment further eliminates hydrogen embrittlement due to atomic-level deposition and stress relief, while also enhancing interfacial cohesion [46,52,53]. The resulting microstructure, which exhibits a weight loss rate of 0.162% at 600 °C, outperforms arc-sprayed coatings in resisting the penetration of corrosive media during cavitation tests [23,33]. Furthermore, compared to processes such as anodizing or chromating, the autocatalytic reaction of ENP does not require an external power source or harmful electrolytes, aligning with the trend towards green manufacturing [27,44,46].

Although the ENP + heat treatment technology demonstrates significant advantages, its application still faces certain limitations. Firstly, the electroplating process necessitates precise control of plating bath parameters (such as pH value and stabilizer concentration) to avoid coating defects, which imposes high demands on process stability. Secondly, compared to rapid processing technologies, such as laser cladding or arc spraying, this process requires a longer heat treatment time (2 h in this study), potentially reducing overall production efficiency. Furthermore, while the anti-cavitation performance of the ENP coating significantly improves after heat treatment at 600 °C (with an improvement rate of 80.1%), its maximum hardness (895 HV) remains lower than that of laser-clad ceramic coatings (>1200 HV) [21], thus limiting its application potential in extreme wear environments. Therefore, the choice of technology requires comprehensive consideration of actual needs: ENP + heat treatment, with its high cost-effectiveness, strong environmental compatibility, and excellent coating uniformity, is particularly suitable for the surface protection of precision components, such as cylinder liners, whereas laser alloying or arc spraying technologies are more appropriate for harsh working conditions that demand ultra-hard coatings or rapid processing. These conclusions align with the discussion on the industrial applicability of ENP technology in existing studies [51,54], further confirming its unique value as a comprehensive solution in cavitation-sensitive systems.

## 4. Conclusions

The present research developed a novel method of ENP plating combined with high-temperature heat-treatment surface modification to resist cavitation. An ultrasonic cavitation test was employed to simulate cavitation working conditions, and the surface morphology, elemental composition, weight loss (weight loss rate), and surface microhardness of unplated, Ni-P-plated, and heat-treated Ni-P-plated layers were analyzed.

(1) The morphology of the ENP plating comprises a microcrystalline structure with fine and dense surface grains and a flat and smooth surface. After heat treatment, the plating structure is gradually transformed from amorphous to crystalline, with many interparticle bonding surfaces and fine grain boundaries, which improve the toughness and plasticity of the plating layer and act as a buffer for the impact stress of bubbles, decreasing the cavitation fatigue crack expansion rate.

(2) As the heat-treatment temperature increased, the loss and loss rate of the plated specimen initially rose to 0.0376 g and 0.162% at 600 °C and then decreased. Meanwhile, the maximum values for the Ni-P plated layer without heat treatment were 0.1895 g and 0.813%, respectively. The heat-treated weight loss at 600 °C was 80.2% lower than that under the plated condition, and the cavitation resistance was improved by 80.1%. The heat-treated Ni-P plating exhibited shallower cavitation pits, and the initial cavitation point lagged behind that of the plated specimens.

(3) The microhardness of the heat-treated Ni-P plating was always much higher than the surface hardness of the unplated and non-heat-treated plated specimens, and its maximum hardness of 895 HV was 588 HV higher than the maximum hardness of the unplated specimen (306 HV), constituting an improvement rate of 65.8%, and higher than the maximum hardness of the non-heat-treated plated specimen of 335 HV, constituting an improvement rate of 62.6%.

Compared to alternative surface modification techniques, the ENP combined with heat treatment method presents distinct advantages in terms of cavitation resistance. While laser alloying can achieve higher hardness levels (>1200 HV) through the formation of intermetallic phases, it is often associated with high costs and thermal stresses, and it struggles with geometric complexity [17,18,19]. Similarly, arc-sprayed coatings allow for rapid thick-layer deposition but are hindered by issues related to porosity and poor corrosion resistance [23,29]. In contrast, ENP offers uniform, dense coatings characterized by excellent adhesion and adaptability to complex geometries, although it does have limitations regarding thickness and processing time [46,47].

In the future, to better integrate laboratory simulations with real-world applications, it is essential to optimize the parameters of the ultrasonic cavitation simulator (e.g., amplitude and frequency) using field vibration data obtained from engine tests. This approach ensures greater consistency with actual operating conditions. Furthermore, conducting cavitation tests within a controlled temperature range (e.g., 50–100 °C) is crucial for elucidating the interactions between coolant thermodynamics and surface treatments, thereby providing significant insights for optimizing performance in dynamic thermal environments. These studies aim to enhance both theoretical knowledge and the practical application of anti-cavitation surface engineering techniques.

## Figures and Tables

**Table 1:** Chemical composition (wt%) of the cylinder liners.

Element	C	S	Si	P	Mn	Cr	Cu	Ni	Mo
Content	2.93	0.036	2.25	0.061	0.481	0.052	0.347	1.261	0.863

**Table 2:** Weight data of specimens with Ni-P composite plating pre- and post-cavitation.

Temperature (℃)	Pre-Test Weight W_0_ (g)	Post-Test Weight W_1_ (g)	Weight Loss W_0_-W_1_ (g)	Weight Loss Rate (%)	Cavitation Depth (μm)	t100 (min)
100	23.2538	23.1306	0.1231	0.529	23.969	3750
200	23.2528	23.1225	0.1303	0.561	25.379	3542
300	23.2531	23.0374	0.1156	0.497	22.519	3992
400	23.2382	23.1050	0.1332	0.573	25.956	3463
500	23.2203	23.1523	0.0680	0.293	13.255	6782
600	23.1612	23.1236	0.0376	0.162	7.345	12,240
Plated	23.2967	23.1072	0.1895	0.813	36.831	2441

## Data Availability

The original contributions presented in this study are included in the article. Further inquiries can be directed to the corresponding author.

## Nomenclature

EDS	Energy dispersive spectrometer	TDC	Top dead center
SEM	Scanning electron microscope	BDC	Bottom dead center
